# Lumpy skin disease of cattle: an emerging problem in the Sultanate of Oman

**DOI:** 10.1007/s11250-013-0483-3

**Published:** 2013-10-07

**Authors:** Mohamed Hassan Tageldin, David Brian Wallace, Gertruida Hermanna Gerdes, John Fraser Putterill, Roelf Rudolph Greyling, Maanda Noaxe Phosiwa, Rashied Mohammed Al Busaidy, Sultan Issa Al Ismaaily

**Affiliations:** 1 Department of Animal and Veterinary Sciences, College of Agricultural and Marine Sciences, Sultan Qaboos University, P.O Box 34, PC 123 Muscat, Sultanate of Oman; 2 ARC-Onderstepoort Veterinary Institute, Private Bag X5, Onderstepoort, 0110 South Africa; 3 Department of Veterinary Tropical Diseases, Faculty of Veterinary Science, University of Pretoria, Private Bag X4, Onderstepoort, 0110 South Africa; 4 Ministry of Agriculture, P.O Box 467, PC 100 Muscat, Sultanate of Oman; 5 Deltamune, 248 Jean Ave, Centurion, 0157 South Africa; 6 Churchill Mansions, Observatory, 7925 Cape Town, South Africa

**Keywords:** Histopathology, LSD, PCR, Serum neutralization, Sultanate of Oman, TEM

## Abstract

Lumpy skin disease (LSD) is a highly infectious disease of cattle caused by a virus belonging to the *Capripoxvirus* genus of the family *Poxviridae*. The purpose of this study is to place on record the first confirmation of LSD in the Sultanate. The disease was diagnosed and confirmed using polymerase chain reaction, histopathology, transmission electron microscopy and serum neutralization testing. The epizootic occurred in 2009 involving a large number of animals and covering a wide area including Nezwa, Alqabel, Sohar, Saham and Burimi. Morbidity and mortality rates of 29.7 and 26.3 %, and 13.6 and 15.4 % were observed at Nezwa and Sohar, respectively. The clinical signs were much more severe in Holstein–Friesian cattle compared to indigenous breeds and were characterized by multiple skin nodules covering the neck, back, perineum, tail, limbs and genital organs. Affected animals also exhibited lameness, emaciation and cessation of milk production. Oedema of limbs and brisket, and superficial lymph node enlargement were highly prominent. It is not known from where the virus originated, or how it spread to the Sultanate. The disease has become endemic in the country and is liable to extend to other Gulf Cooperation Council Countries and cause a pandemic. It is of major concern to the Omani dairy industry. Due to the widespread presence of screw worm, serious economic losses can follow outbreaks.

## Introduction

Lumpy skin disease (LSD) is a serious disease of cattle caused by lumpy skin disease virus (type strain, Neethling), which, together with sheep and goat poxviruses, constitute the *Capripoxvirus* genus of the *Poxviridae* family (Davies and Otema 1981; Woods 1988). The disease is characterized by large skin nodules covering all parts of the body, fever, enlarged lymph nodes, nasal discharge and lachrymation, but the severity of clinical signs is highly variable (Davies 1991a). LSD causes significant economic losses due to permanent hide damage. Temporary or permanent infertility may occur in cows and bulls (Tuppurainen and Oura 2011). It leads to reduced milk yield and sometimes death due to secondary bacterial infections (Chihota et al. 2003). In addition, it disrupts the trade in cattle and their products from LSD endemic countries (Babiuk et al. 2008). LSD was initially restricted to countries in sub-Saharan Africa, but from 1984 to 1988, there were unconfirmed reports of the disease in cattle in Oman and Kuwait (House et al. 1990; Kumar 2011). In 1988, it was confirmed in Egypt where it subsequently became enzootic (Ali et al. 1990; House et al. 1990; Davies 1991a). The virus then apparently spread by insect transmission from Egypt to Israel in 1989 (Davies 1991b), causing this disease to occur in a number of dairy herds, with a second outbreak occurring in dairy herds in 2006 (Brenner et al. 2006). In both outbreaks, the implementation of vaccination, strict quarantine measures and slaughter policies was successful in eradicating the disease (Davies 1991a; Yeruham et al. 1995; Brenner et al. 2006). LSD was also suspected in Saudi Arabia in 1989 in Arabian oryx (*Oryx leucoryx*) (Greth et al. 1992).

Clinical cases suggestive of LSD was observed in the Sultanate of Oman in 2009 (Kumar 2011). It was characterized by the appearance of multiple skin nodules covering the entire body associated with the persistent high fever and depression.

A comprehensive clinical report and follow-up treatment on the outbreak in one dairy herd in the Batinah (Sohar) region is available (Kumar 2011). Our report describes the first confirmation of LSD in Oman from Sohar and Nezwa outbreaks and highlights the associated pathological features.

## Materials and methods

### Clinical history and sample collection

In April 2009, a severe disease of cattle resembling LSD was reported from Nezwa (Interior), Alqabel (Eastern), Sohar, Saham (Batinah) and Burimi regions. The outbreaks involved seven herds (64 North Oman, Jersey and cross-bred cattle) and one herd (3,300 Holstein–Friesian dairy cows) at Nezwa and Sohar, respectively. Samples were collected from 22 and 38 cows from Nezwa and Sohar, respectively. Skin biopsies were collected for virus isolation, polymerase chain reaction (PCR), negative staining for transmission electron microscopy and histopathology. Sera were collected for serum neutralization testing (Beard et al. 2010) and necropsies were performed on two dead Holstein–Friesian animals. Biopsies and tissues collected at necropsy were fixed in 10 % buffered formalin, processed, sectioned and stained with either haematoxylin and eosin or phloxine–tartrazine stain (Bancroft and Gamble 2008).

### Sample preparation for electron microscopic examination

Small tissue sections were excised from visible lesions on the affected tissue and homogenised using a mortar and pestle in sterile double-distilled water (ddH_2_O). The suspension was centrifuged at low speed (1,000 × *g*) for 5 min to remove coarse debris. The supernatant was further centrifuged at 10,000 × *g* for 20 min and the supernatant fraction discarded. The pellet was gently washed twice with ddH_2_O and suspended in phosphotungstic acid (pH 6.4). This suspension was then applied dropwise to a Formvar-coated copper grid, allowed to dry and viewed at 80 kV using a Jeol JEM-1200 transmission electron microscope (Japan).

### Sample preparation for polymerase chain reaction analysis

A thin tissue section removed from each sample using sterile technique was chopped into 5 mm^3^ cubes and transferred to a separate mortar. Sterile phosphate-buffered saline (PBS) (2 ml) was added and the pieces were ground with a pestle in carborundum powder. The mixtures were then transferred to Eppendorf tubes and allowed to stand for 3 min to precipitate large detritus. The supernatants were transferred to new Eppendorf tubes and sonicated using a Sonorex TK52 waterbath sonicator (Bandelin, Germany) at 35 kHz for 10 min. The mixtures were subsequently vortexed and centrifuged at 2,000 rpm (358 × *g*) for 2 min in an Avanti 30 Beckman benchtop centrifuge (Beckman, USA). The supernatants were transferred to new Eppendorf tubes and centrifuged at 16,000 rpm (22,897 × *g*) for 15 min to pellet the viral particles. The supernatants were discarded and the virus-containing pellets resuspended in 200 μl PBS for DNA extraction using a MagNA Pure LC Total Nucleic Acid Isolation Kit (Roche, Germany) on a MagNA Pure LC Instrument (Roche, Germany) according to the manufacturer's instructions.

### PCR conditions

The primers were designed from sequence data derived from the South African Onderstepoort vaccine strain and Warm baths field isolate of LSDV (Genbank accession numbers AF409138 and AF409137, respectively) (Kara et al. 2003). Primer pair 1, consisting of primer DW-TK (5′- GCC GAT AAC ATA TAT AGA CCC −3′) and primer OP49 (5′- GTG CTA TCT AGT GCA GCT AT −3′), is used to amplify a 434-bp LSDV genomic fragment between positions 56698–57132, and primer pair 2, consisting of primer L132F (5′- CAC TTC CCT TTT AAG C −3′) and primer L132R (5′- CAT TCT ACA ATC TCC ATG CG −3′), amplifies a 492-bp fragment between genomic positions 119801–120292. The PCRs were performed using an Eppendorf Master Cycler® gradient thermo cycler (Merck, Germany) and 25 μl reaction volumes consisting of 2.5 μl 10× PCR buffer (containing 20 mM MgCl_2_) (Takara Biomedical, Japan), 2 μl 2.5 mM dNTPs (Takara Biomedical, Japan), 0.25 U Taq DNA polymerase (TaKaRa Ex Taq™, Takara Biomedical, Japan), 20 nmoles of each primer (Gibco-Brl, Scotland), template DNA (~0.1 ng) and sterile ddH_2_O. Template DNA was denatured initially for 90 s at 95 °C, followed by 35 cycles of denaturation (45 s at 95 °C), primer annealing (45 s at 56 °C) and strand extension (60 s at 72 °C), ending with a final strand extension step for 7 min at 72 °C. These conditions were used for both primer pairs.

## Results

### Distribution and clinical signs

The morbidity and mortality rates of 29.7 and 26.3 %, and 13.6 and 15.4 % at Nezwa and Sohar, respectively. At Alqabel and Burimi, 17 and 19 cattle were infected with mortality rates of 5.9 and 5.3 %, respectively (data on morbidity rates were unavailable). At Sohar, 85 % of infected animals either died as the result of complications or were culled due to weakness and low milk productivity. Holstein–Friesian cows showed more severe clinical signs compared to local indigenous cattle. The clinical signs were characterized by nodules ranging in size from several millimetres to 2–5 cm in diameter on various parts of the body, particularly on the head, neck, back, perineum, udder, testicles, tail and limbs. Clinical signs also included salivation, lameness, severe emaciation, cessation of milk production and death. Oedema of the limbs, brisket and superficial lymph node enlargement were very prominent. Conjunctivitis, keratitis and corneal opacity were frequently seen. Necropsy showed nodules on the dorsum of the tongue. The skin nodules involved the epidermis, dermis, subcutaneous tissue and the musculature.

At the onset of the outbreak at Sohar, all non-infected cattle (2,851 animals) were vaccinated with live attenuated sheep and goat pox vaccine (Kenya strain, KS1). Five percent of these animals developed clinical signs of the disease.

### Histopathology and TEM

**Fig. 1:**
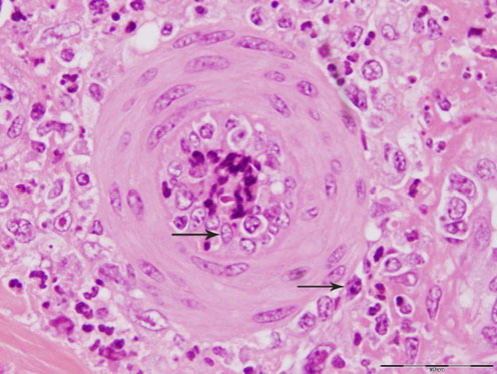
A small artery in the vicinity of a skin nodule. Note the vasculitis indicated by the presence of inflammatory cells inside and around blood vessels (*arrows*)

**Fig. 2:**
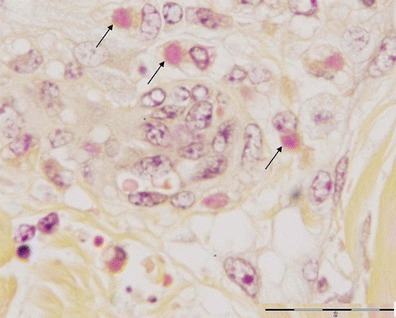
Mononuclear cells displaying intracytoplasmic inclusions (*arrows*)

**Fig. 3:**
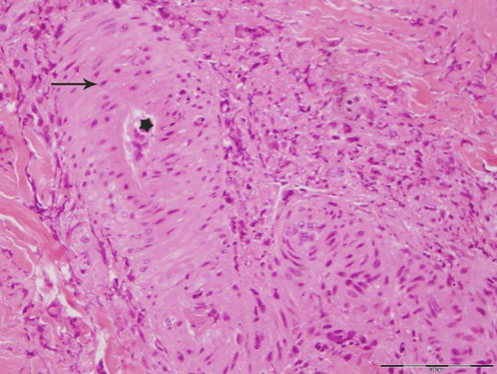
A small artery showing marked thickening of the tunica media (*arrow*), as well as a narrow lumen (*star*)

The skin lesions were characterized by multifocal necrosis and inflammatory infiltration in the epidermis and/or dermis. In some lesions, the necrosis and inflammatory responses were limited to the dermis, while the overlying epidermis was largely intact. These lesions were observed near sweat glands, hair follicles or sebaceous glands. The inflammatory cells observed were mainly lymphocytes, with low numbers of macrophages and occasionally eosinophils. These cells were particularly prominent around blood vessels adjoining the necrotic lesions (Fig. 1). Intracytoplasmic inclusions were observed in mononucleated cells (Fig. 2). The vascular changes were very prominent and included vasculitis, perivasculitis and perivascular necrosis with concomitant thrombosis. Some arteries depicted thickening of tunica media associated with narrowing of the lumen (Fig. 3).

**Fig. 4:**
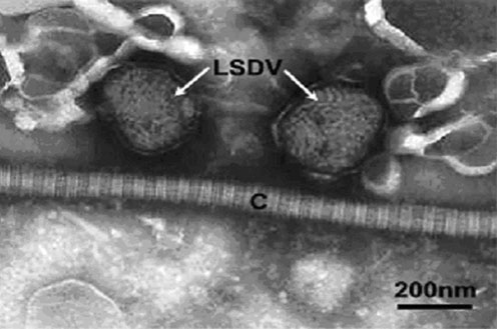
Transmission electron micrograph of two negatively PTA-stained LSDV particles indicated (*arrows*) in close association with a collagen fibre (*C*). The particles show a typical thread-like structure on their surface and typical “brick-shaped” morphology (*arrows*)

Electron micrograph images of negatively stained preparations showed high densities of typical poxvirus particles (Fig. 4).

### Serology

Sera were collected from Nizwa from infected Jersey, North Oman and North Oman crossed with Jersey cattle, whereas the healthy in contact with infected animals were North Oman cattle and North Oman cattle crossed with Jersey (Table 1). Sera collected from infected Holstein–Friesian cattle were from Sohar. All sera samples tested from infected cattle were positive for antibodies to LSD, whereas those from the healthy in contact with infected animals did not show any detectable antibodies (Table 1).

**Table 1:** LSD serum neutralization testing for local, cross and exotic breeds

No of serum sample	Area	Neutralization	Animal breed	Animal health
1	Nezwa	Positive	Jersey	Infected
3	Nezwa	Positive	North Oman cattle × Jersey	Infected
7	Nezwa	Positive	North Oman cattle	Infected
1	Nezwa	Negative	North Oman cattle	Healthy-in contact
3	Nezwa	Negative	North Oman × Jersey	Healthy-in contact
19	Sohar	Positive	Holstein–Friesian	Infected

Positive antibodies detected range from 1:4 to 1:24. Negative means no antibodies detected

### PCR analysis

The two primer pairs used for virus identification are homologous to regions of the LSDV thymidine kinase (TK) and ORF132 genes, respectively. The TK gene is highly conserved among the capripoxviruses and thus primer pair 1 also binds to the TK genes of sheep pox and goat pox viruses. However, LSDV ORF132 is unique to LSDV and thus primer pair 2 only binds to LSDV DNA (data not shown).

**Fig. 5:**
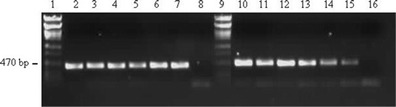
Agarose gel (1 %) electrophoresis separation of PCR amplification products of Oman LSD samples. Note: DNA was stained using ethidium bromide and viewed and photographed under a UV light source. *Lanes 1 and 9*, Phage lambda PstI marker DNA; *lanes 2*–*8*, PCR products generated using primer pair 1; *lanes 10*–*16*, PCR products generated using primer pair 2. *Lane 2*, Oman 1; *lane 3*, Oman 2; *lane 4*, Oman 3; *lane 5*, Oman 4; *lane 6*, positive control (LD18 LSDV field isolate); *lane 7*, positive control (LSDV Onderstepoort vaccine); *lane 8*, sterile water negative control; *lane 10*, Oman 1; *lane 11*, Oman 2; *lane 12*, Oman 3; *lane 13*, Oman 4; *lane 14*, positive control (LD18 LSDV field isolate); *lane 15*, positive control (LSDV Onderstepoort vaccine); *lane 16*, negative control (sterile water)

The results of PCR amplification of DNA extracted from the Oman cattle samples using both primer pairs are shown in Fig. 5. Amplification products of the expected sizes for LSDV were obtained for all the samples, including the positive LSDV controls, when analysed using agarose gel electrophoresis and ethidium bromide staining under UV light, thus indicating that the samples contained a strain of LSDV.

## Discussion

Based on the clinical signs, histopathology, PCR, electron microscopy and serum neutralization testing, LSD was diagnosed in Omani cattle in the epizootic of 2009. This represents the first confirmation for the presence of LSD in the Sultanate of Oman. The disease is considered a transboundary animal disease due to its significant impact on trade and food security, and its ability to spread to other countries (Rossiter and Al Hammadi 2009). The real danger of the disease lies in the fact that it has continued to spread, extending its range to include all of Africa, with infrequent appearances in the Middle East (Woods 1988). However, LSD has been eradicated from Israel at a high cost through rapid diagnosis, slaughtering of all diseased and in-contact cattle and small ruminants, and vaccination (Davies 1991b). The LSD epizootic in the Oman involved a large number of animals and a wide area, including Eastern, Interior and Batinah regions. It is unclear how the disease is maintained during inter-epidemic periods (Hunter and Wallace 2001). The virus is thought to persist by unapparent infection cycling in cattle or in old lesions, and low-level persistence in wildlife cannot be excluded (Davies 1991b; Hunter and Wallace 2001). The epizootics observed in the Oman have shown more variation in morbidity and mortality and in the rate at which they spread, as have been previously reported (Ali et al.1990; House et al.1990; Davies, 1991a, b). Such differences are thought to reflect the transmission efficiency and population densities of potential vector populations (Davies 1991b). Holstein–Friesian cattle showed more severe skin lesions compared to the local breeds. It is not known what genetic factors influence the disease severity (Babiuk et al. 2008); however, it is claimed that very young calves, lactating cows and animals suffering from malnutrition generally develop the most severe infections, probably due to impaired cellular immunity (Hunter and Wallace 2001). High ambient temperatures, coupled with farming practices to produce high milk yields, could be deemed to stress the animals and contribute to the severity of the disease in Holstein–Friesian cattle.

The crossbred and North Oman cattle that were in contact with the infected animals remained healthy and failed to develop detectable neutralizing antibodies indicating that direct contact plays little role in the transmission of LSD (Davies 1991a; Carn and Kitching 1995; Babiuk et al. 2008).

The usual quarantine methods, including animal isolation and vaccination, were ineffective control measures in preventing the spread of LSD within the herd at the Sohar farm. This could have been due to the animals already incubating the virus, thus making it too late for the vaccine to afford protection (Hunter and Wallace 2001) or, as observed in Israeli dairy herds (Brenner et al. 2006), the possibility exists that the Kenya sheep and goat pox vaccine is underattenuated, causing clinical disease in a significant proportion of vaccinated animals.

The skin is the most susceptible organ for virus replication (Babiuk et al. 2008), explaining the high virus levels detected in the skin by electron microscopy. In contrast to previous findings (Prozesky and Barnard 1982; House et al. 1990), our skin histopathology results did not reveal the presence of microvesicles, hyperkeratosis or acanthosis. In addition, the presence of eosinophils and the prominent vascular changes were more conspicuous than those reported by Prozesky and Barnard (1982). This discrepancy could be attributed to differences in the stage of disease progression at which the samples were collected or virus strain differences.

All animals displaying clinical signs related to LSD, including the local crossbred and Holstein–Friesian cattle, revealed low levels of neutralizing antibody. This could be attributed to the collection of sera at the early stage of infection when antibody levels are still low or due to the presumed immunosuppressive properties of the virus (Kara et al. 2003).

Due to the widespread presence of screw worm (*Chrysomya bezziana*) in the Sultanate, the danger of co-existence of LSD with myiasis is another problem that could have a serious impact on the economy of the country.

The precise origin of the LSD virus responsible for the outbreak in the Sultanate is unknown. However, it is possible that the disease was introduced into the country by infected cattle imported from the African Horn countries including Somalia and Djibouti. The uncontrolled movement of infected animals may be a factor which increases the hazard of disease spreading to various regions of the Sultanate. Circumstantial evidence suggests that future epizootics are likely to occur. It is feared that the disease could cross boundaries into neighbouring countries or spread fairly rapidly throughout the region and become a pandemic. A new outbreak of LSD in Israel in July 2012 (ProMed report no. 20120728.1218484) attests to the fact that LSD is now firmly entrenched within the Middle East and widespread preventative measures should become routine practice.
